# Management of an Intruded Tooth and Adjacent Tooth Showing External Resorption as a Late Complication of Dental Injury: Three-Year Follow-Up

**DOI:** 10.1155/2015/741687

**Published:** 2015-02-23

**Authors:** Ülkü Şermet Elbay, Mesut Elbay, Emine Kaya, Alper Sinanoglu

**Affiliations:** ^1^Department of Pediatric Dentistry, Faculty of Dentistry, Kocaeli University, 41190 Kocaeli, Turkey; ^2^Department of Oral and Maxillofacial Radiology, Faculty of Dentistry, Kocaeli University, 41190 Kocaeli, Turkey

## Abstract

Treatment and prognosis of intrusive luxation can vary depending on the age of the patient, type of dentition, stage of root development, and time and severity of the trauma. Some studies have demonstrated that intrusions of up to 3.0 mm have an excellent prognosis, whereas teeth with severe intrusion or teeth that are intruded more than 6.0 mm present an unfavorable prognosis because of the occurrence of inflammatory resorption and pulp necrosis. The aim of this case report is to present an 11-year-old male patient with complete intrusion of the permanent maxillary left lateral incisor, associated with the adjacent central tooth presenting external resorption, treated by immediate surgical repositioning and root canal treatment with a favorable prognosis. After long-term (3-year) clinical and radiographic follow-up, the teeth appeared normal and the patient was pleased with the outcome.

## 1. Introduction

Traumatic injuries of the anterior teeth in children have a psychological impact on both the parents and the child, especially if the injury affects the permanent dentition. Having untreated fractured teeth has been directly related to the emotional state and appearance of children [1, 2]. Berger et al. [3] presented negative effects of severe dentoalveolar trauma on the quality of life of children and parents by using a child oral health quality of life questionnaire in their study.

Dental trauma can result in a number of injuries involving the tooth and the supporting structures. Six types of luxation and seven types of tooth fracture have been described and are used to classify traumatic dental injuries [4]. Intrusive luxation is one of the most severe types of dental traumatic injuries and is defined as a dislocation of the tooth in an apical direction into the socket [5, 6]. Intrusion rarely occurs in the permanent dentition when compared with other types of luxation injuries. It comprises 0.5–1.9% of all traumatic injuries in the permanent dentition and 5%–12% of dental luxations [5–7].

The treatment and prognosis of intrusive luxation can vary depending on the age of the patient, type of dentition, stage of root development, and time and severity of the trauma [8, 9]. There is no agreement in the literature for the ideal treatment for intruded permanent teeth after trauma [9]. A variety of treatment modalities are suggested to manage intrusive luxation. One option is to allow the tooth to reerupt on its own. Another treatment is to reposition the tooth using orthodontic forces. Immediate surgical repositioning has also been recommended [2, 10–14]. Waiting for spontaneous reeruption is indicated for immature permanent teeth, due to their high potential for eruption and pulp/periodontal repair. If the tooth is grossly intruded (>7 mm) and root formation is complete, then surgical or orthodontic repositioning, followed by splinting for 2–3 weeks, is indicated [15].

Luxation injuries represent a very complex wound that involves disruption of the marginal gingival seal, alveolar bone, periodontal ligament fibers, cementum, and the neurovascular supply to the pulp; they can result in severely compromised healing and possible complications [16]. Possible complications are pulp necrosis, pulp canal obliteration, root resorption, and loss of marginal alveolar bone [4, 16]. The preferred treatment method and long-term consideration should minimize these complications after traumatic injuries. Because the majority of dental injuries involve the anterior teeth and usually affect a single tooth, other types of trauma predict multiple injuries [17] and some of them may not be detected at the time of the initial injury and may cause delayed complications. This risk emphasizes the need for careful monitoring of pulpal responses to traumatic injuries and radiographic changes.

This report presents a case with severe intrusive luxation of a maxillary lateral incisor and external root resorption of the adjacent central incisor of which injury was not detected at the time of the initial trauma but as a late complication.

## 2. Case Report

An 11-year-old male patient, with a dental injury after falling on the edge of a bed, presented to the clinic of Pediatric Dentistry Department of Kocaeli University, 2 hours after the accident. He complained of pain and sensitivity of the left upper lateral tooth and the gingiva around it. His mother was upset and worried about his condition. This was the first dental trauma experience for him and his medical history was unremarkable.

After the patient's general, medical, and trauma history was recorded, a clinical examination was completed. The extraoral examination for hard and soft tissues revealed no signs of injury, such as swelling, colour changes of the skin, or face and head asymmetry. No abnormalities were observed when the facial bones and mandible were palpated to assess the mouth opening.

**Figure 1:**
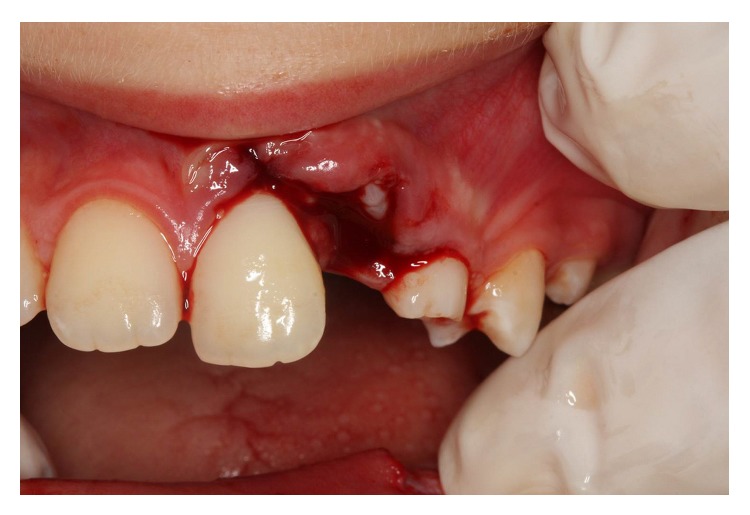
Intraoral appearance of the intruded left permanent upper lateral incisor.

The intraoral clinical examination revealed the complete intrusion of the permanent maxillary left lateral incisor, as well as a gingival laceration (Figure 1). There was no evidence of traumatic injury to any other teeth. Additionally, the maxillary left and right central incisors tested as being vital according to percussion and electrical pulp tester.

**Figure 2:**
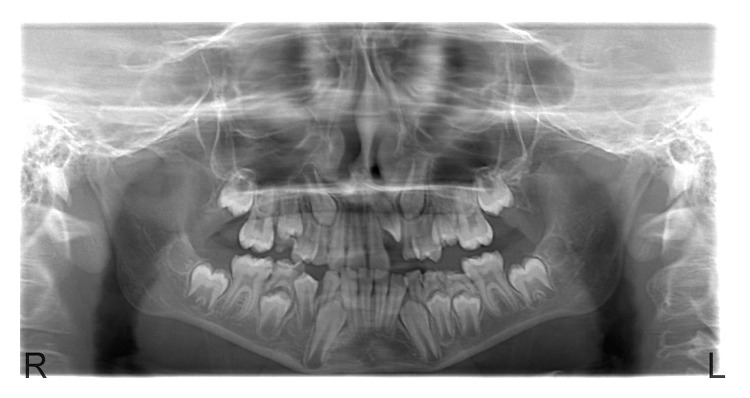
Orthopantomographic view after trauma.

The radiographic examination revealed that the left maxillary lateral incisor was completely and severely intruded (Figure 2). The root of the intruded tooth was completely developed. The periodontal space surrounding the intruded tooth was lost, and no root or bone fractures were detected. Also, there was no radiographic evidence of injury to any other teeth.

**Figure 3:**
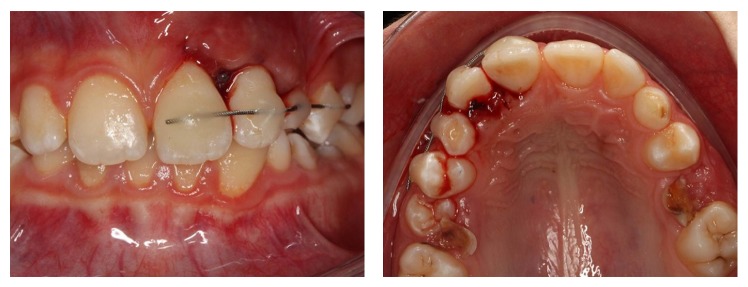
Left upper lateral incisor after replacement and splinting.

Due to the severity of the intrusion and apical development of the root, an immediate surgical repositioning of the lateral incisor was planned. After administering local anesthesia, the intruded tooth was luxated gently with an elevator to minimize cell damage to the periodontal ligament and to the cementum. The intruded tooth was repositioned to its original place. The traumatized lateral incisor was splinted from the central incisor to the ipsilateral first premolar using 0.5 mm stainless-steel multistranded flexible orthodontic wire and an acid etch-composite resin technique (Figure 3). Due to the partial resorption of the root of the primary canine tooth, the first premolar was included in the splint. Endodontic treatment of the lateral incisor tooth was started at the first visit. After accessing, extirpating, and instrumenting, calcium hydroxide that was mixed with sterile saline was placed into the canal. The access cavity was sealed with glass ionomer cement. The patient was asked to maintain good oral hygiene.

**Figure 4:**
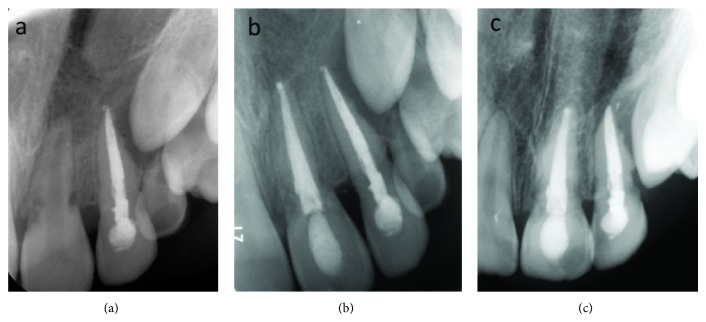
(a) Left upper central incisor with external root resorption at the 6-month follow-up, (b) periapical radiograph at the 12-month follow-up, and (c) periapical radiograph at the 24-month follow-up.

The patient was reexamined 3 weeks after the trauma and the splint was removed. The patient was recalled every month for six months. At each recall visit, the calcium hydroxide dressing was changed and mobility, percussion, palpation, and vitality tests were performed and radiographic images of the intruded and adjacent incisors were obtained. At the 6th month visit, the intruded tooth was completely asymptomatic, so its treatment was finished with obturation of the root canals using gutta-percha and sealing the coronal access using resin composite. At the same visit, the radiographic examination revealed external root resorption of the adjacent central incisor as a late complication of the luxation injury (Figure 4(a)). Additionally, the central incisor was tested as nonvital. Therefore, endodontic treatment of the central incisor was initiated at this visit. The same endodontic treatment protocol was carried out as was used for the lateral incisor. After 6 months, the external root resorption was observed to have stopped. The treatment of the central incisor was finished with obturation of the root canals using gutta-percha and sealing of the coronal access using resin composite.

**Figure 5:**
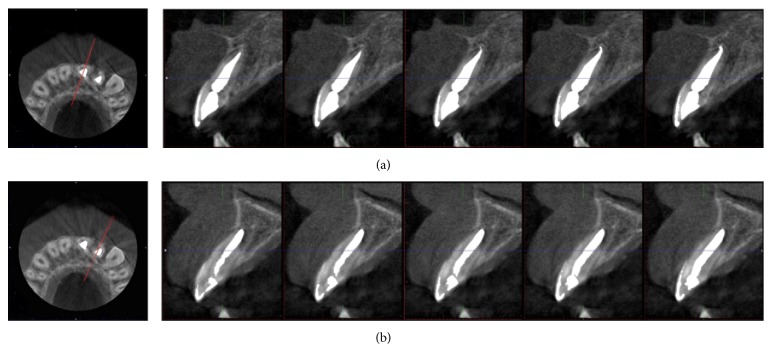
CBCT images of the left upper lateral and central incisor at the 3-year follow-up.

**Figure 6:**
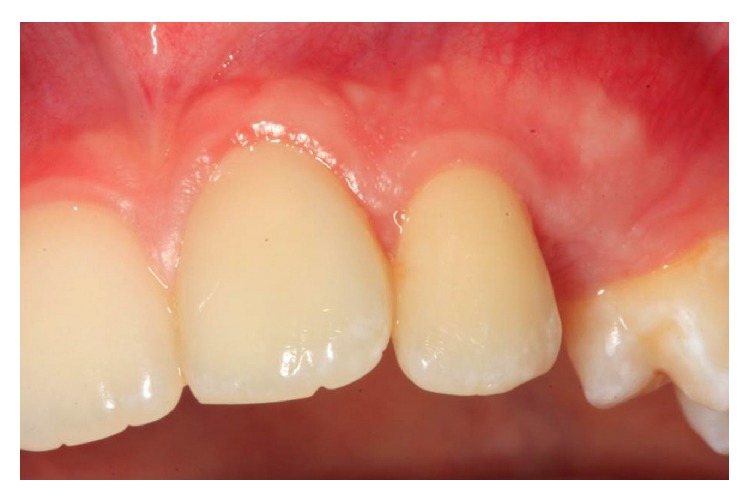
Intraoral appearance of the patient at the 3-year follow-up.

In the present case, the teeth were stable and the patient was completely asymptomatic 12 months postoperatively (Figure 4(b)). Also 24-month radiographic examination showed that there were no signs to indicate resorption or infection (Figure 4(c)). At the 3-year follow-up examination, a small volume CBCT scan of the maxilla was performed (Planmeca ProMax 3D Max, Planmeca, Finland) at 96.0 kV, 11.0 mA, and 12.3 s, to examine the traumatized teeth. CBCT scan was set at the smallest field of view protocol (5 cm × 5.5 cm) of the device, targeting specifically the maxillary incisor area. Images were analyzed by viewing software (Planmeca Romexis, Planmeca, Finland). The maxillary lateral and central incisors were found to have remained in position and there were no signs of periapical lesions or resorption (Figure 5). Also, the clinical findings demonstrated that the chosen clinical protocol was successful as both teeth remained asymptomatic; there was no gingival inflammation or mobility (Figure 6). In addition, the patient was satisfied with his appearance and approved of his smile.

## 3. Discussion

Intrusive luxation is one of the most severe types of dental traumatic injuries [6]. Intrusion is considered to have the poorest prognosis of all dental traumas because it significantly damages the periodontal ligament (PDL), the pulp, or the alveolar bone [16].

The effect on the PDL can be observed in cases where root resorptions take place. These have been identified as repair-related (surface), infection-related (inflammatory), and ankylosis-related (replacement) resorption [18, 19]. Repair-related resorption is a transient process involving small areas on the root surface following luxation injuries. As long as conditions are right for healing, that is, absence of bacteria in the root canal, this type of resorption is reversible [19]. A very aggressive type of injury-related resorption is an inflammatory resorption. It may be related to pulpal or periodontal infection. For pulpal infection, following injury to the precementum or predentin, infected dentinal tubules may stimulate the inflammatory process with osteoclastic activity in the periradicular tissues or in pulp. For periodontal infection, bacteria from the periodontal sulcus may penetrate in dentinal tubules, coronal to the epithelial attachment, and exit apical to the epithelial attachment without penetrating the pulpal space [19–21]. A relatively slower, but not necessarily more benign, resorptive process is ankylosis-related resorption. This type of resorption is associated with extensive trauma to the PDL resulting in loss of vitality of the cells and extensive damage to the cementum. Because of the lack of a protective covering, the root cementum is exposed to osteoclasts that mistake cementum for bone and proceed to replace the cementum and dentin with new bone, resulting in a fusing of the bone and the tooth [19–21]. In this case, while ankylosis-related resorption was not seen and only repair-related resorption was seen for the left maxillary lateral incisor despite the severe intrusion, infection-related resorption was seen for the adjacent central incisor probably due to the periodontal or pulpal infection of the tooth after injury.

Considering the very high risk of loss of pulpal vitality and root resorption, root canal treatment is often indicated in cases of moderate to severe intrusion [20]. The recommended time to start root canal treatment is approximately two weeks after the injury. In cases of severe intrusion, this early endodontic therapy is facilitated by rapid surgical repositioning. In the presence of inflammatory root resorption, nonsetting calcium hydroxide paste should be used and appropriately replaced in the canal until root resorption is controlled, prior to obturation [20–23]. As mentioned above, due to the severity of intrusion and apical development of the root in the presented case, endodontic treatment of the maxillary lateral incisor was started at the first visit, as soon as immediate surgical repositioning occurred. Because luxation is prone to damage the cemental protection of root surfaces, allowing dentinal tubules to become pathways for bacterial toxins within the canal to trigger osteoclastic activity externally, calcium hydroxide was applied for disinfection of dentinal tubules. Because the intruded tooth was not completely asymptomatic at the follow-up visits, the calcium hydroxide dressing was changed until the intruded tooth was completely asymptomatic.

A clinical diagnosis of root resorption is frequently made following an incidental radiographic finding. This is because most cases of resorption remain asymptomatic until it reaches an advanced stage. Hence, the radiographic interpretation of the resorptive process is crucial for the differential diagnosis, treatment, and prognosis of the tooth [24]. In our case, radiographic examination at the 6th month visit revealed external root resorption of the adjacent incisor as a late complication of the luxation injury. Endodontic intervention was required and calcium hydroxide was placed. When the external root resorption had been arrested, the root canal treatment was completed.

Traumatized teeth present a clinical challenge with regard to their diagnosis, treatment planning, and prognosis. Recent developments in imaging systems have enabled clinicians to effectively visualize structural changes. Three-dimensional imaging allows evaluation of the area of interest away from the superimposition surrounding structures [24, 25]. CBCT is an emerging technology which was specifically developed to produce undistorted information with a substantially lower radiation dose when compared to conventional CT [26]. Using small- or limited-volume cone beam-computed tomography imaging techniques produces precise 3D images of the teeth and surrounding dentoalveolar structures. In the present study, for image acquisition, the small field of view protocol was chosen to minimize patient dosage and the radiation beam was collimated to the restricted area. Routine use of CBCT for endodontic purposes was not recommended but it was advised that CBCT might be justifiable in the assessment of dentoalveolar trauma in selected cases, where conventional radiographs provide inadequate information [27]. In our case, CBCT images were evaluated in submillimeter slice thickness in order to assess any resorption process in the labial and the lingual surface of the root from axial images and cross-sectional images, without superimposition of anatomical structures. As mentioned before, CBCT scan was not the image of choice at the first period of the treatment of the present case. It was taken to evaluate the presence of any root resorption after 3 years of follow-up of an intrusion case, associated with the adjacent central tooth presenting external resorption. From this perspective, we do not recommend routine use of CBCT in the pediatric population, regarding that the lower radiation dose in the imaging of children must be a strict consideration [28] in accordance with the general principle of radiology, ALARA (as low as reasonably achievable).

Treatment of the complications of luxation injuries is often complex, time consuming, expensive, and unpredictable. These late healing complications highlight the need for regular long-term follow-up to aid in their detection and treatment in all cases of luxation. Also, new extraoral imaging systems may be of assistance in detecting complications earlier when compared with periapical radiographs, and the true size, extent, nature, and location of periapical and resorptive lesions can be more accurately assessed.
